# Sex‐ and Laterality‐Specific Associations Between Serum Uric Acid‐to‐Creatinine Ratio and Gait Parameters in Parkinson's Disease

**DOI:** 10.1002/brb3.71737

**Published:** 2026-09-21

**Authors:** Hongwei Guo, Kangmei Shao, Yujie Bu, Wenjuan Li, Jianping Hu, Yanfang Zhang, Jianxiong Li

**Affiliations:** ^1^ The Second Clinical Medical School Lanzhou University Lanzhou Gansu China; ^2^ Department of Neurology Lanzhou University Second Hospital Lanzhou Gansu China

**Keywords:** gait, Parkinson's disease, motor laterality, Parkinson's disease, uric acid‐to‐creatinine ratio

## Abstract

**Objective:**

Uric acid is believed to potentially have neuroprotective effects in Parkinson's disease (PD), but its link to gait performance and potential moderation by sex or motor symptom laterality remains unclear. This study explored whether the association between the serum uric acid‐to‐creatinine (UA/Cr) ratio and spatial gait parameters varies by sex and the dominant side of motor symptoms in PD patients.

**Methods:**

Seventy‐seven patients with early‐stage PD were assessed. Concurrently, spatial gait parameters including step height and step length were measured, as well as serum UA/Cr levels. Conditional process analysis (PROCESS Model 2) examined whether sex and motor symptom laterality jointly moderated the UA/Cr–gait relationship, controlling for age, body mass index, disease duration, levodopa equivalent dose, and motor severity. Subgroup‐specific conditional effect analyses further clarified these associations.

**Results:**

The dual‐moderation model explained 54.45% of the variance in step height (FDR‐adjusted *p* = 0.0204). Associations between UA/Cr and gait were highly subgroup‐dependent. Men with right‐dominant motor symptoms showed a significant positive relationship between UA/Cr and both step height and step length (*p* < 0.05). Conversely, women with left‐dominant symptoms exhibited a negative association between UA/Cr and step height (*p* = 0.0258). Other sex–laterality subgroups showed no significant associations.

**Conclusion:**

The findings indicate that sex and motor symptom laterality may jointly shape how UA/Cr relates to spatial gait performance in PD. The positive association was most pronounced in men with right‐dominant motor symptoms, while among female patients with left‐ dominant motor symptoms, a relatively weaker negative correlation was observed. Given the exploratory nature and modest sample size, these results should be interpreted cautiously and warrant confirmation in larger, longitudinal studies to clarify subgroup‐specific neuroprotective effects of uric acid.

## Graphical abstract

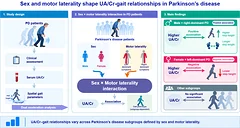

## 1 Introduction

Parkinson's disease (PD) is a common neurodegenerative disorder characterized primarily by bradykinesia, resting tremor, rigidity, and postural and gait abnormalities. Compared with age‐matched healthy individuals, patients with PD commonly show reduced gait speed and shortened step length; however, these changes are not specific to PD (Pistacchi 2017). In contrast, reduced arm swing amplitude and increased motor asymmetry are more characteristic of PD and often appear in the early stage of the disease (Baron et al. 2018; Mirelman et al. 2016). Conventional clinical rating scales can assess overall motor symptoms, but their ability to quantitatively evaluate gait abnormalities is limited (Mirelman et al. 2019). Studies of prodromal PD have shown that mild motor dysfunction may precede the clinical diagnosis of PD (Noyce et al. 2016). Therefore, gait analysis has received increasing attention in the assessment of motor function in PD.

Oxidative stress is considered to play an important role in the onset and progression of PD. In response, researchers have increasingly scrutinized uric acid (UA), a natural endogenous antioxidant, because it may exert neuroprotective effects (Xu et al. 2025). Previous studies have associated elevated serum UA with reduced PD risk (Grażyńska et al. 2021; Wen et al. 2017). Compared with UA alone, the UA/Cr ratio may better reflect overall UA metabolism while accounting for muscle mass and renal function, thus providing additional information for evaluating UA‐clinical phenotype relationships in PD (Zhong et al. 2018; Songsomboon et al. 2020).

Existing studies on UA in PD have mainly focused on clinical subtypes and neuroimaging features. For example, patients with the postural instability and gait difficulty (PIGD) subtype of PD may have lower UA levels and reduced striatal dopamine transporter availability (Huertas et al. 2017), as well as more pronounced substantia nigra hyperechogenicity on ultrasonography (Peng et al. 2025). In addition, lower UA levels have been associated with the occurrence of freezing of gait (Ou et al. 2017). Among patients with PD and freezing of gait, lower UA levels have also been linked to poorer nutritional status (Zhang et al. 2022). Mechanistic studies have further suggested that UA may influence PD‐related pathological changes through multiple pathways (Sheng et al. 2017; Lee et al. 2022; Cipriani et al. 2012). However, studies directly examining how UA or UA/Cr relates to spatiotemporal gait parameters in PD are scarce. These parameters parse motor function into discrete, limb‐specific measures and reveal asymmetrical deficits that global assessments often overlook (Silveira‐Ciola et al. 2026). Therefore, they may serve as digital biomarkers for exploring the relationship between metabolic indicators and motor phenotypes in PD (Mancini et al. 2025).

The UA‐PD relationship is neither uniform across sexes nor independent of motor symptom asymmetry. Men with higher UA appear less vulnerable to PD onset (Cortese et al. 2018) and maintain better attention and executive performance (Lee et al. 2018). In women, the pattern differs: elevated UA tracks with greater dopamine transporter uptake in the globus pallidus (Oh et al. 2020), and inosine trials suggest females experience slower progression alongside higher plasma antioxidant capacity (Schwarzschild et al. 2019). Because PD motor symptoms are inherently lateralized, the dominant side introduces another layer of complexity. The impact of UA on non‐motor and cognitive outcomes appears to hinge on whether symptoms are left‐ or right‐dominant, and this lateralized effect itself varies by sex (Constantin et al. 2023). Consequently, treating sex and motor asymmetry as mere background noise risks obscuring genuine metabolic‐clinical associations; they are more accurately understood as joint moderators that actively reshape how UA relates to the PD phenotype.

Despite these findings, it remains unclear whether the association between UA/Cr and gait parameters is influenced by sex and motor symptom‐dominant side. We therefore examined how serum UA/Cr relates to discrete spatiotemporal gait parameters in PD, and whether this relationship is moderated by the dominant side of symptoms and by sex. Treating gait as a set of spatiotemporal signatures rather than a global summary allowed us to evaluate whether UA/Cr acts as a subgroup‐specific metabolic biomarker. We hypothesized that the relationship between UA/Cr and spatiotemporal gait parameters would be moderated by symptom‐dominant side and sex. Specifically, we expected that higher UA/Cr would be associated with better spatial gait performance in patients with right‐dominant symptoms (Schwarzschild et al. 2008). Meanwhile, given sex‐related differences in UA metabolism and antioxidant capacity, the association between UA/Cr and gait parameters may be more pronounced in female patients (Silveira‐Ciola et al. 2026; Nicoletti et al. 2017).

## 2 Materials and Methods

### 2.1 Study Design and Participant Selection

This retrospective cross‐sectional study was conducted in the Department of Neurology at Lanzhou University Second Hospital. Patients with PD who received clinical care between April 2025 and April 2026 were retrospectively identified from the electronic medical record system and the institutional gait assessment database. Eligibility was determined according to predefined inclusion and exclusion criteria. Demographic information, clinical assessment results, laboratory findings, and gait‐related measures were subsequently extracted from the corresponding records. To ensure the completeness and reliability of the dataset, the final analysis included only patients with complete clinical information, available serum UA and creatinine measurements, and valid gait assessment data. The study protocol was approved by the Ethics Committee of Lanzhou University Second Hospital (approval no. 2026A‐756). Given the retrospective design and the use of routinely collected clinical data, the requirement for informed consent was waived.

### 2.2 Inclusion and Exclusion Criteria

Patients were eligible for inclusion if they met all of the following criteria: (Pistacchi 2017) a diagnosis of PD established according to the Movement Disorder Society clinical diagnostic criteria (Baron et al. 2018), documented serum UA and creatinine measurements (Mirelman et al. 2016), standardized gait assessment data collected during the study period, and (Mirelman et al. 2019) complete demographic and clinical information required for the analysis. Patients were excluded if they had other neurological disorders that could substantially affect gait, such as stroke, normal pressure hydrocephalus, or peripheral neuropathy; severe osteoarticular disease, trauma, or a history of lower‐limb surgery that could interfere with normal walking; severe cardiopulmonary dysfunction that prevented completion of the gait test; gout, severe renal insufficiency, or use of medications that could markedly affect UA metabolism; or (Noyce et al. 2016) incomplete clinical, laboratory, or gait assessment data.

### 2.3 Clinical and Laboratory Data Collection

Patient data were collected, including age, sex, disease duration, serum uric acid‐to‐creatinine (UA/Cr) ratio, Hoehn–Yahr (H–Y) stage, Movement Disorder Society‐Unified Parkinson's Disease Rating Scale Part III (MDS‐UPDRS‐III) score, tremor and non‐tremor scores, and levodopa equivalent daily dose (LEDD). These data were extracted from electronic medical records.

Fasting venous blood samples were collected within 3 days before or after the gait assessment for measurement of serum UA and creatinine. Both biomarkers were analyzed in the central laboratory of Lanzhou University Second Hospital using a Roche Cobas 8000 modular analyzer series. Serum UA and creatinine concentrations were recorded in standard laboratory units, after which the UA/Cr ratio was calculated.

Clinical and laboratory variables were extracted using a standardized data collection form and cross‐checked against the electronic medical records. Patients were excluded if data were missing for the primary exposure, gait outcomes, or key covariates included in the analysis.

### 2.4 Assessment of Motor Symptoms and Motor Laterality

Motor symptoms of PD were assessed using the MDS‐UPDRS‐III. Motor symptom asymmetry was evaluated based on the difference between bilateral item scores of the MDS‐UPDRS‐III. Items 3.3, 3.4, 3.5, 3.6, 3.7, 3.8, 3.15, 3.16, and 3.17 were used to calculate the symptom asymmetry index (SAI), using the following formula:

$$\mathrm{SAI}\;=\left(\mathrm{left}-\mathrm{right}\right)/\left(\mathrm{left}\;+\;\mathrm{right}\right)$$

Positive SAI values indicated greater left‐sided motor symptom severity, whereas negative SAI values indicated greater right‐sided motor symptom severity. Items with bilateral scores of zero were treated as symmetrical. Motor symptom laterality (MSL) was defined according to the SAI. Patients with PD were subsequently classified as having left‐dominant motor symptoms (LPD) or right‐dominant motor symptoms (RPD). To quantify the degree of clinical laterality, the absolute value of the SAI (ABS‐SAI) was used. Four participants with symmetrical motor symptoms were excluded from subgroup analyses because they could not be assigned to either the LPD or RPD group.

### 2.5 Gait Assessment

All patients underwent gait assessment in the ON medication state. Spatiotemporal gait parameters were collected using the ReadyGo‐1‐2 balance testing system (Suzhou Ruiyi Medical Technology Co., Ltd., Suzhou, China). During the assessment, participants were instructed to walk naturally at their usual comfortable speed along a 3‐m walkway according to standardized instructions and completed three round‐trip walking trials. The system automatically recorded spatiotemporal gait parameters for each trial, and the mean value of the three trials was used for subsequent analyses. The gait parameters included in the analysis were gait speed, step length, step height, double‐support phase percentage, and turning time. For gait parameters with left‐ and right‐side measurements, bilateral mean values were used in the analysis to reflect overall gait performance and reduce the influence of side‐specific measurement variability.

### 2.6 Grouping Strategy

Four subgroups were defined according to MSL and sex. Patients with left‐dominant motor symptoms (LPD) were divided into male patients with LPD (LPDm; *n* = 19) and female patients with LPD (LPDf; *n* = 20). Patients with right‐dominant motor symptoms (RPD) were divided into male patients with RPD (RPDm; *n* = 21) and female patients with RPD (RPDf; *n* = 17).

### 2.7 Statistical Analysis

To examine whether the relationship between the serum UA/Cr ratio and spatiotemporal gait parameters was moderated by MSL and sex, dual‐moderation analyses were performed using the PROCESS macro developed by Hayes. In the primary analysis, PROCESS Model 2 was applied, with UA/Cr entered as the focal predictor, motor symptom‐dominant side and sex entered as two parallel moderators, and each spatiotemporal gait parameter entered as the dependent variable. Age, body mass index (BMI), levodopa equivalent daily dose (LEDD), disease duration, and MDS‐UPDRS‐III score were included as covariates.

Because motor symptom asymmetry may involve both direction and magnitude, additional sensitivity analyses were performed for step height and step length. First, the absolute value of the symptom asymmetry index (ABS‐SAI), as a continuous indicator of the degree of motor asymmetry, was entered into the model together with sex as parallel moderators. Second, motor symptom‐dominant side and ABS‐SAI were simultaneously entered as moderators, with sex additionally adjusted as a covariate, to evaluate whether the moderating effect of motor symptom‐dominant side remained stable after accounting for the degree of asymmetry.

UA/Cr and continuous moderator variables were mean‐centered before analysis. All regression models were estimated using HC3 heteroscedasticity‐consistent standard errors to reduce the potential influence of heteroscedasticity on parameter estimation and significance testing. To account for multiple testing, Benjamini–Hochberg false discovery rate correction was applied within each gait parameter across interaction terms and conditional effects. Given the exploratory cross‐sectional design and limited subgroup sample sizes, subgroup‐specific findings were interpreted as hypothesis‐generating. All statistical analyses were conducted using SPSS version 27.0. Figures were prepared using GraphPad Prism (version 11.0).

## 3 Results

### 3.1 Baseline Characteristics

Table 1 summarizes baseline characteristics across the four subgroups. The groups were well matched in age, BMI, disease duration, LEDD, motor severity, clinical asymmetry, and gait parameters.

**TABLE 1:** Demographic, clinical, biochemical, and gait characteristics according to motor symptom laterality and sex.

Variable	LPDm (*n* = 19)	LPDf (*n* = 20)	RPDm (*n* = 21)	RPDf (*n* = 17)	*p* value
Age, years	68.00 (58.00, 75.00)	67.00 (59.25, 71.00)	63.00 (59.00, 70.50)	66.00 (58.50, 74.00)	0.645
BMI, kg/m^2^	23.81 (22.64, 25.88)	23.09 (21.92, 27.00)	23.73 (21.95, 24.55)	23.63 (22.27, 24.61)	0.710
Disease duration, years	2.00 (2.00, 5.00)	2.00 (1.00, 4.00)	3.00 (1.00, 4.50)	2.00 (1.00, 5.00)	0.807
LEDD, mg/day	337.50 (337.50, 437.50)	337.50 (300.00, 346.88)	337.50 (300.00, 387.50)	337.50 (337.50, 468.75)	0.274
MDS‐UPDRS‐III	19.00 (11.00, 28.00)	24.00 (15.50, 31.75)	19.00 (9.50, 30.50)	19.00 (10.50, 25.00)	0.600
H‐Y	1.50 (1.00, 2.00)	2.00 (1.00, 2.00)	1.00 (1.00, 1.75)	1.00 (1.00, 2.00)	0.305
UA, µmol/L	298.00 (264.00, 381.00)	314.00 (249.25, 367.25)	245.00 (222.50, 261.00)	238.00 (199.00, 272.00)	< 0.001
Cr, µmol/L	73.10 (64.60, 78.40)	69.95 (62.18, 85.65)	56.50 (51.95, 61.60)	57.60 (50.50, 62.40)	< 0.001
UA /Cr	4.02 (3.67, 4.79)	4.30 (3.68, 5.04)	4.13 (3.84, 4.88)	3.99 (3.61, 4.97)	0.988
ABS‐SAI	36.84 (13.04, 75.00)	51.19 (28.79, 86.11)	36.84 (20.83, 72.22)	71.43 (33.33, 92.31)	0.307
Gait speed, m/s	0.82 (0.55, 0.95)	0.82 (0.70, 0.95)	0.83 (0.51, 0.95)	0.72 (0.61, 0.98)	0.919
Step length, m	0.47 (0.39, 0.57)	0.48 (0.41, 0.56)	0.46 (0.35, 0.53)	0.43 (0.32, 0.52)	0.589
Step height, m	0.11 (0.10, 0.13)	0.12 (0.11, 0.14)	0.11 (0.08, 0.13)	0.11 (0.08, 0.12)	0.185
Double support phase, %	38.48 (35.29, 41.83)	38.26 (35.39, 40.27)	36.92 (34.56, 42.62)	37.69 (35.06, 42.01)	0.890
Turning time, s	1.87 (1.37, 2.85)	1.88 (1.45, 2.45)	1.63 (1.20, 2.09)	1.63 (1.38, 2.47)	0.667

*Note*: Data are presented as median (interquartile range, Q1–Q3). Group differences were assessed using the Kruskal–Wallis test. A two‐sided *p* value < 0.05 was considered statistically significant.

Abbreviations: ABS‐SAI, absolute symptom asymmetry index; BMI, body mass index; Cr, creatinine; f, female; H–Y, Hoehn and Yahr stage; LEDD, levodopa equivalent daily dose; LPD, left‐dominant motor symptoms; m, male; MDS‐UPDRS‐III, Movement Disorder Society–Unified Parkinson's Disease Rating Scale Part III; RPD, right‐dominant motor symptoms; UA, uric acid; UA/Cr, serum uric acid‐to‐creatinine ratio.

Serum UA and creatinine levels differed across groups, whereas UA/Cr showed no significant differences among the four subgroups.

### 3.2 Moderating Effects of MSL and Sex

Dual‐moderation analyses were performed to examine whether MSL and sex moderated the association between UA/Cr and spatial gait parameters.

For step height, the model captured 54.45% of the variance. Both two‐way interactions—UA/Cr × motor symptom‐dominant side and UA/Cr × sex—emerged as significant, indicating that the slope of UA/Cr on step height changes direction or magnitude depending on which side dominates the patient's symptoms and on whether the patient is male or female. The joint test of the two interaction terms was significant, *F* (2, 66) = 4.587, uncorrected *p* = 0.0136, FDR‐adjusted *p* = 0.0204.

The pattern for step length was broadly similar but statistically weaker. The model accounted for 57.70% of the variance. The UA/Cr × motor symptom‐dominant side interaction crossed the uncorrected significance threshold, *B* = 0.0562, *p* = 0.0473, while the UA/Cr × sex interaction fell just short of conventional significance, *B* = −0.0518, *p* = 0.0616. Their joint test likewise hovered at the margin, *F* (2, 66) = 2.775, *p* = 0.0696. Detailed results are provided in Table 2.

**TABLE 2:** Moderating effects of motor symptom laterality and sex on the association between UA/Cr and spatial gait parameters.

		*B*	SE(HC3)	95% CI	*F*	*p* value	FDR‐adjusted *p*
Step height	UA/Cr× MSL	0.0154	0.0055	0.0045 to 0.0264		0.0065	0.0195
	UA/Cr × Sex	−0.0127	0.0055	−0.0238 to −0.0017		0.0246	0.0246
	Joint test (H_0_: both = 0)				4.587	0.0136	0.0204
Step length	UA/Cr× MSL	0.0562	0.0278	0.0007 to 0.1117		0.0473	0.0696
	UA/Cr × Sex	−0.0518	0.0272	−0.1061 to 0.0026		0.0616	0.0696
	Joint test				2.775	0.0696	0.0696

*Note*: All models were adjusted for age, body mass index, levodopa equivalent daily dose, disease duration, and MDS‐UPDRS‐III score. UA/Cr was mean‐centered before analysis. Standard errors and confidence intervals were estimated using HC3 heteroscedasticity‐consistent standard errors. The *F* statistic and *p* value for the joint test were obtained from the “BOTH” row in the PROCESS output, testing the null hypothesis that the coefficients of UA/Cr × MSL and UA/Cr × sex were both zero. The degrees of freedom for the joint tests were *df*1 = 2 and *df*2 = 66. *p* values were adjusted within each gait parameter across the two interaction terms and the joint interaction test using the Benjamini–Hochberg false discovery rate method.

**Abbreviations**: CI, confidence interval; FDR, false discovery rate; MSL, motor symptom laterality; UA/Cr, serum uric acid‐to‐creatinine ratio.

### 3.3 Conditional Effects by Sex and MSL

**FIGURE 1:**
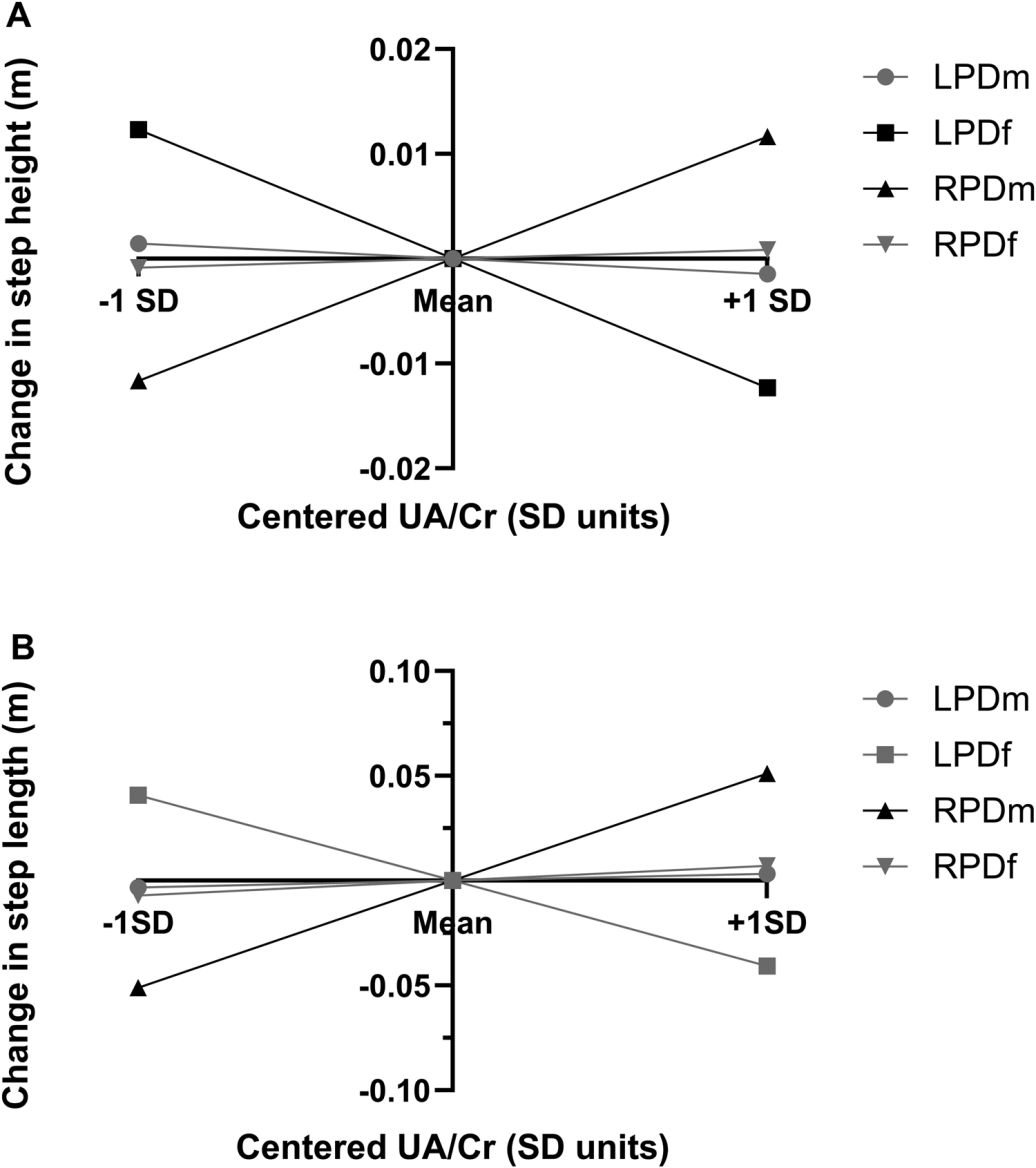
Conditional associations between centered UA/Cr and spatial gait parameters stratified by motor symptom laterality and sex. (A) Conditional associations between centered UA/Cr and step height. (B) conditional associations between centered UA/Cr and step length. Regression lines represent subgroup‐specific conditional effects (simple slopes) estimated from models adjusted for age, BMI, disease duration, LEDD, and MDS‐UPDRS‐III score. HC3 robust standard errors were used. Values represent predicted changes in step height and step length relative to the mean UA/Cr level, calculated at ±1 SD of UA/Cr. Regression coefficients (*B*) and *p* values for simple slopes are provided in Table 3. Black lines and symbols indicate statistically significant associations after FDR correction (adjusted *p* < 0.05), whereas gray lines and symbols indicate non‐significant associations. BMI, body mass index; LEDD, levodopa equivalent daily dose; LPDf, female patients with left‐dominant motor symptoms; LPDm, male patients with left‐dominant motor symptoms; MDS‐UPDRS‐III, Movement Disorder Society–Unified Parkinson's Disease Rating Scale Part III; RPDf, female patients with right‐dominant motor symptoms; RPDm, male patients with right‐dominant motor symptoms; UA/Cr, serum uric acid‐to‐creatinine ratio.

We then probed the conditional effects within each sex‐by‐laterality subgroup to see where the interaction terms actually mattered. As illustrated in Figure 1 and summarized in Table 3, the associations between UA/Cr and spatial gait parameters varied across sex‐by‐laterality subgroups. Drilling down to step height, higher UA/Cr was associated with greater step height only in men with right‐dominant symptoms, *B* = 0.0137, 95% CI [0.0032, 0.0242], *p* = 0.0258. Conversely, among women with left‐dominant symptoms, higher UA/Cr was associated with lower step height, *B* = −0.0145, 95% CI [−0.0257, −0.0032], FDR‐adjusted *p* = 0.0258. The remaining two subgroups—LPDm and RPDf—showed no detectable link (both *p* > 0.05). Step length followed a narrower pattern: the association surfaced only in RPDm, *B* = 0.0601, 95% CI [0.0179, 0.1023], FDR‐adjusted *p* = 0.0240, and vanished in the other three subgroups (all *p* > 0.05; see Table 3 for full conditional estimates).

**TABLE 3:** Conditional effects of UA/Cr on spatial gait parameters by motor symptom laterality and sex.

Subgroup	Step height *B*	SE (HC3)	95% CI	FDR‐adjusted *p* value	Step length *B*	SE (HC3)	95% CI	FDR‐adjusted *p* value
LPDm	−0.0017	0.0036	−0.0090 to 0.0055	0.8197	0.0039	0.0194	−0.0349 to 0.0426	0.8422
LPDf	−0.0145	0.0057	−0.0257 to −0.0032	0.0258	−0.0479	0.0323	−0.1123 to 0.0166	0.2854
RPDm	0.0137	0.0053	0.0032 to 0.0242	0.0258	0.0601	0.0211	0.0179 to 0.1023	0.0240
RPDf	0.0010	0.0043	−0.0076 to 0.0096	0.8197	0.0083	0.0229	−0.0374 to 0.0540	0.8422

*Note*: All models were adjusted for age, body mass index, disease duration, levodopa equivalent daily dose, and MDS‐UPDRS‐III score. UA/Cr was mean‐centered before analysis. HC3 heteroscedasticity‐consistent standard errors were used in all regression models. *p* values for conditional effects were adjusted within each gait parameter across the four‐motor symptom laterality‐by‐sex subgroups using the Benjamini–Hochberg false discovery rate method.

**Abbreviations**: CI, confidence interval; f, female; FDR, false discovery rate; LPD, left‐dominant motor symptoms; m, male; RPD, right‐dominant motor symptoms; UA/Cr, serum uric acid‐to‐creatinine ratio.

### 3.4 Sensitivity Analyses Using the Magnitude of Motor Symptom Asymmetry

We next asked whether forcing laterality into a binary split had obscured a graded effect. Replacing the dominant‐side dummy variable with continuous ABS‐SAI and entering it alongside sex as parallel moderators, we again found a significant joint effect on step height, *F* (2, 66) = 4.0249, uncorrected *p* = 0.0224, FDR‐adjusted *p* = 0.0298 (Table S1). For step length, the joint effect was attenuated but still survived FDR correction, *F* (2, 66) = 3.1768, uncorrected *p* = 0.0482, FDR‐adjusted *p* = 0.0482. Probing these interactions, men with pronounced asymmetry (high ABS‐SAI) showed a marginal positive UA/Cr–step height slope, whereas women with minimal asymmetry (low ABS‐SAI) showed a marginal negative slope (Table S2).

### 3.5 Sensitivity Model Including both Laterality Direction and Asymmetry Magnitude

To disentangle directional laterality from asymmetry magnitude, we entered both motor symptom‐dominant side and ABS‐SAI as moderators in the same model, controlling for sex. The UA/Cr × dominant side interaction for step height retained a marginal signal, *B* = 0.0133, FDR‐adjusted *p* = 0.0669. The UA/Cr × ABS‐SAI interaction was weaker and did not reach significance, suggesting that the left–right distinction carries more explanatory weight than the continuous degree of asymmetry itself. Detailed results are provided in Table S3.

## 4 Discussion

We set out to explore whether the serum UA/Cr ratio relates to spatial gait parameters in PD and, more importantly, whether this relationship shifts depending on which side of the body bears the heavier motor burden and on whether the patient is male or female. The dual‐moderation models pointed to exactly this kind of conditional dependency: the slope of UA/Cr on gait performance appeared to change across clinical subgroups, with the step height model offering the most suggestive pattern. Step height, also referred to as toe clearance, is a clinically relevant spatial gait parameter that reflects the strategy used to elevate the foot during walking. It has been proposed as an informative indicator of gait impairment and fall susceptibility in patients with PD (di Biase et al. 2020). In the present analysis, the joint test further indicated that the two interaction terms contributed collectively to the step‐height model. Sensitivity analyses using continuous ABS‐SAI also identified moderation signals for spatial gait parameters, although these findings were not entirely consistent across models. Probing the conditional effects exposed a striking subgroup specificity: higher UA/Cr predicted better spatial gait performance in some subgroups but worse in others, with the direction and magnitude of the association appearing to hinge on the particular combination of sex and laterality (Table 3 and Figure 1). Taken together, these preliminary findings suggest that the association between UA/Cr and spatial gait performance may be heterogeneous across PD phenotypes rather than uniform across all patients. Subtle gait abnormalities can occur early in PD and may not be fully captured by global motor scales; quantitative spatial gait measures may therefore provide complementary phenotypic information when examining relationships between peripheral metabolic markers and motor function (Zanardi et al. 2021; Wu et al. 2021).

The observed subgroup‐specific patterns should be interpreted with caution. UA sits at the end of the purine metabolic chain and doubles as one of the body's own antioxidants (Waring et al. 2003). Accordingly, UA has been investigated as a potential biomarker of oxidative stress‐related vulnerability and disease progression in PD (Schwarzschild et al. 2008). Men and women diverge in how they synthesize, retain, and clear UA, and these metabolic differences appear to carry clinical weight: in men, higher circulating UA has been linked to slower disease progression (Schwarzschild et al. 2008; Ascherio et al. 2009), whereas women may follow a different trajectory altogether. Women generally have lower serum urate levels than men, which may be related to sex hormones and sex‐specific differences in renal urate handling (Halperin Kuhns and Woodward 2020). The positive association between UA/Cr and gait performance observed in men with right‐dominant symptoms appears broadly consistent with previous reports of sex‐specific urate‐related effects. By contrast, the inverse association identified in women, particularly those with left‐dominant symptoms, is less straightforward to interpret and may partly reflect sampling variation, residual confounding, or biological factors not captured in the present analysis. UA/Cr itself is a composite signal: it folds together UA, creatinine, and by extension, muscle bulk and renal clearance efficiency (Songsomboon et al. 2020). Differences in body composition and renal handling may have contributed to the subgroup‐specific associations observed in this study. In particular, the stronger association between UA/Cr and gait performance in men could partly reflect their generally greater muscle mass or sex‐related differences in renal urate excretion. These interpretations remain tentative, however, and do not readily explain the inverse association observed in the LPDf subgroup, which warrants replication in an independent cohort. Motor laterality is a relatively stable clinical feature of PD (Lee et al. 1995; Miller‐Patterson et al. 2018) and has been linked to variation in cognitive and neuropsychiatric phenotypes (Katzen et al. 2006), raising the possibility that symptom laterality reflects broader disease heterogeneity. Whether such heterogeneity underlies the observed associations between UA/Cr and gait remains unclear. The direction and degree of motor asymmetry may also represent partly distinct clinical dimensions: laterality direction showed a trend toward statistical significance, whereas the interaction involving asymmetry magnitude was less evident. Overall, these potential mechanisms are speculative and should be examined in larger studies incorporating more detailed assessments of renal function, body composition, and hormonal status.

Gait abnormalities and motor asymmetry may emerge early in PD and evolve as the disease progresses (Dluzen 2000), supporting the potential value of quantitative gait measures for identifying subtle phenotypic differences. From a clinical perspective, UA/Cr is inexpensive and readily available in routine practice. However, the present findings do not support its use as a diagnostic, prognostic, or treatment‐guiding biomarker. Rather, UA/Cr may have potential as an auxiliary marker for risk stratification (Mallet et al. 2022; Toś et al. 2025). When considered together with sex and MSL, it may provide additional information for identifying PD subgroups that are potentially more vulnerable to gait impairment. These findings further suggest that the relationship between UA/Cr and gait should not necessarily be interpreted uniformly across all patients, but may depend partly on sex and the laterality of motor symptoms. If confirmed in prospective studies, UA/Cr could complement quantitative gait assessment by contributing to the elucidation of phenotypic heterogeneity and by helping to generate hypotheses about which patient subgroups may warrant closer longitudinal monitoring of gait function. Importantly, the SURE‐PD3 trial showed that inosine‐induced elevation of serum urate did not slow PD progression and increased the risk of kidney stones (Parkinson Study Group SURE‐PD3 Investigators et al. 2021). Therefore, the associations observed in the present study should not be interpreted as evidence that increasing serum urate would improve gait performance or alter disease progression. Future validation should be conducted in adequately powered, multicenter prospective cohorts with prespecified sex‐ and laterality‐based analyses, repeated UA/Cr and gait assessments, and more comprehensive evaluations of renal function, body composition, hormonal status, and multiple gait domains including variability, coordination, dynamic balance, and freezing of gait. Before any clinical application, the incremental value of UA/Cr beyond demographics, motor severity, renal function, and body composition must be demonstrated, and clinically meaningful thresholds would need to be established and externally validated.

This study has several limitations. First, the findings were based on a cross‐sectional or single‐time‐point model, and therefore the causal direction between UA/Cr and gait parameters cannot be determined. Second, UA/Cr is not equivalent to serum UA alone and may be influenced by muscle mass, renal function, diet, medication use, and body composition. Third, although the models were adjusted for age, BMI, LEDD, disease duration, and MDS‐UPDRS‐III score, unmeasured confounders may still exist, including renal function indices, dietary purine intake, estrogen status, cardiometabolic risk factors, and physical activity levels. Fourth, multiple moderation models and conditional effect analyses were performed, increasing the risk of chance findings; subgroup‐specific results, especially the negative associations in left‐dominant women and women with low ABS‐SAI, should be considered exploratory. Fifth, the subgroup analyses may have been limited by sample size, especially after stratification by motor symptom‐dominant side and sex, which may have reduced statistical power. Sixth, this study used PROCESS Model 2 to test the parallel moderating effects of ABS‐SAI and sex, but did not directly examine the three‐way interaction among UA/Cr, ABS‐SAI, and sex. Therefore, whether the degree of asymmetry and sex exert an interactive joint moderating effect requires further investigation.

In conclusion, the association between UA/Cr and spatial gait performance appeared to differ across PD subgroups defined by sex and MSL. However, the inconsistency across models and the small subgroup sample sizes limit the robustness of these observations. The findings should therefore be regarded as hypothesis‐generating and require confirmation in prespecified, adequately powered longitudinal studies before their potential clinical relevance can be determined.

## 5 Conclusion

This exploratory cross‐sectional study suggests that the association between UA/Cr and spatial gait parameters in patients with PD may vary according to sex and MSL. Higher UA/Cr was associated with better spatial gait performance, particularly step height and step length, mainly in male patients with right‐dominant motor symptoms. In contrast, female patients did not show a comparable favorable association pattern, and inverse associations were observed in specific female subgroups. These findings suggest that UA/Cr should not be interpreted as a uniform protective marker across all patients with PD, but may instead represent a composite metabolic correlate whose relationship with gait may differ according to sex and MSL. Further validation of these subgroup‐specific findings in larger prospective longitudinal cohorts is warranted.

## Author Contributions

All authors contributed to the study conception and design. Data analysis was performed by **Hongwei Guo**. The first draft of the manuscript was written by Hongwei Guo, and all authors commented on previous versions of the manuscript. All authors read and approved the final manuscript.

## Funding

This work was supported by the Natural Science Foundation of Gansu Province (Grant No. 26JRRA815). The funding source provided research platform support for the Parkinson's disease clinical cohort but had no role in the study design, data collection and analysis, decision to publish, or preparation of the manuscript.

## Ethics Statement

This study was approved by the Ethics Committee of Lanzhou University Second Hospital (approval number: 2026A‐756).

## Conflicts of Interest

The authors declare no conflicts of interest.

## Supporting information




**Supplementary Information**: brb371737‐sup‐0001‐SuppMat.docx

## Data Availability

The datasets used and/or analyzed during the current study are available from the corresponding authors on reasonable request.
