# Supplementary material for: Sex‐ and Laterality‐Specific Associations Between Serum Uric Acid‐to‐Creatinine Ratio and Gait Parameters in Parkinson's Disease
Source: Brain Behav. 2026 Sep 21;16(9):e71737. doi: 10.1002/brb3.71737 (PMC13594765; doi:10.1002/brb3.71737)
Supplement: Supplementary file 1 [file BRB3-16-e71737-s001.docx]

Supplementary Table S1. Sensitivity analysis of the moderating effects of ABS-SAI and sex on the association between UA/Cr and spatial gait parameters.

|  |  | *B* | SE(HC3) | 95% CI | *F* | *P* | FDR-adjusted *P* |
| --- | --- | --- | --- | --- | --- | --- | --- |
| Step height | UA/Cr × ABS-SAI | 0.0002 | 0.0001 | 0.0000 to 0.0004 |  | 0.0206 | 0.0298 |
|  | UA/Cr × Sex | -0.0128 | 0.0058 | -0.0244 to -0.0013 |  | 0.0298 | 0.0298 |
|  | Joint test |  |  |  | *F* (2,66) =4.0249 | 0.0224 | 0.0298 |
| Step length | UA/Cr × ABS-SAI | 0.0010 | 0.0004 | 0.0001 to 0.0019 |  | 0.0229 | 0.0482 |
|  | UA/Cr × Sex | -0.0546 | 0.0260 | -0.1064 to -0.0027 |  | 0.0395 | 0.0482 |
|  | Joint test |  |  |  | *F* (2,66) =3.1768 | 0.0482 | 0.0482 |

Note. Values are presented as unstandardized regression coefficients unless otherwise indicated. Sensitivity analyses were performed using ABS-SAI and sex as parallel moderators. All models were adjusted for age, body mass index, levodopa equivalent daily dose, disease duration, and MDS-UPDRS III score. UA/Cr and ABS-SAI were mean-centered before model estimation. Standard errors and 95% confidence intervals were calculated using HC3 heteroscedasticity-consistent estimation. The joint test evaluates the combined contribution of the UA/Cr × ABS-SAI and UA/Cr × sex interaction terms.

Abbreviations: ABS-SAI, absolute symptom asymmetry index; CI, confidence interval; HC3, heteroscedasticity-consistent standard error estimator; SE, standard error; UA/Cr, serum uric acid-to-creatinine ratio.

Supplementary Table S2. Conditional effects of UA/Cr on spatial gait parameters at different levels of ABS-SAI and sex.

|  | ABS-SAI level | Sex | *B* | SE(HC3) | 95% CI | FDR-adjusted *P* |
| --- | --- | --- | --- | --- | --- | --- |
| Step height | Low ABS-SAI, −1 SD | Male | -0.0016 | 0.0041 | -0.0098 to 0.0065 | 0.834 |
|  | Low ABS -SAI, −1 SD | Female | -0.0145 | 0.0057 | -0.0259 to -0.0030 | 0.062 |
|  | Mean ABS -SAI | Male | 0.0055 | 0.0037 | -0.0018 to 0.0128 | 0.209 |
|  | Mean ABS -SAI | Female | -0.0074 | 0.0043 | -0.0160 to 0.0013 | 0.190 |
|  | High ABS -SAI, +1 SD | Male | 0.0126 | 0.0053 | 0.0020 to 0.0232 | 0.062 |
|  | High ABS -SAI, +1 SD | Female | -0.0003 | 0.0048 | -0.0098 to 0.0092 | 0.955 |
| Step length | Low ABS -SAI, −1 SD | Male | -0.0014 | 0.0164 | -0.0343 to 0.0313 | 0.927 |
|  | Low ABS -SAI, −1 SD | Female | -0.0562 | 0.0256 | -0.1072 to -0.0050 | 0.096 |
|  | Mean ABS -SAI | Male | 0.0304 | 0.0178 | -0.0051 to 0.0660 | 0.184 |
|  | Mean ABS -SAI | Female | -0.0243 | 0.0174 | -0.0589 to 0.0107 | 0.256 |
|  | High ABS -SAI, +1 SD | Male | 0.0623 | 0.0272 | 0.0081 to 0.1167 | 0.096 |
|  | High ABS -SAI, +1 SD | Female | 0.0075 | 0.0181 | -0.0284 to 0.0441 | 0.801 |

Note. Unless stated otherwise, model specifications follow Supplementary Table S1. This table presents conditional effects of UA/Cr at specific combinations of ABS-SAI and sex. Low and high ABS-SAI represent 1 SD below and above the mean, respectively. *P* values were adjusted within each gait parameter across the six ABS-SAI-by-sex conditions using the Benjamini-Hochberg false discovery rate method.

Abbreviations: SD, standard deviation. For other abbreviations, see Supplementary Table S1.

Supplementary Table S3. Sensitivity analysis of the moderating effects of symptom laterality and ABS-SAI on the association between UA/Cr and spatial gait parameters

|  | *B* | SE(HC3) | 95% CI | *F* | *P* | FDR-adjusted *P* |
| --- | --- | --- | --- | --- | --- | --- |
| UA/Cr × MSL | 0.0133 | 0.0057 | 0.0020 to 0.0247 |  | 0.0223 | 0.0669 |
| UA/Cr × ABS-SAI | 0.0001 | 0.0001 | 0.0000 to 0.0003 |  | 0.0922 | 0.0922 |
| Joint test |  |  |  | *F* (2,65) =3.237 | 0.0457 | 0.0686 |
| UA/Cr × MSL | 0.0458 | 0.0266 | -0.0074 to 0.0990 |  | 0.0904 | 0.0904 |
| UA/Cr × ABS-SAI | 0.0007 | 0.0004 | -0.0001 to 0.0015 |  | 0.0861 | 0. 0904 |
| Joint test |  |  |  | *F* (2,65) =2.605 | 0.0816 | 0. 0904 |

Note. Unless stated otherwise, model specifications follow Supplementary Table S1. In this model, motor symptom laterality and ABS-SAI served as moderators, with sex included as a covariate. The joint test captures the combined contribution of the UA/Cr × symptom laterality and UA/Cr × ABS-SAI interaction terms. FDR-adjusted *P* were calculated using the Benjamini-Hochberg procedure separately for each dependent variable (step height and step length).

Abbreviations: MSL, motor symptom laterality. For other abbreviations, see Supplementary Table S1.
